# A new Progressive Management Pathway for improving seaweed biosecurity

**DOI:** 10.1038/s41467-022-34783-8

**Published:** 2022-12-01

**Authors:** Elizabeth J. Cottier-Cook, Jennefe P. Cabarubias, Janina Brakel, Juliet Brodie, Alejandro H. Buschmann, Iona Campbell, Alan T. Critchley, Chad L. Hewitt, Jie Huang, Anicia Q. Hurtado, Cicilia S. B. Kambey, Phaik Eem Lim, Tao Liu, Jonalyn P. Mateo, Flower E. Msuya, Zizhong Qi, Louise Shaxson, Grant D. Stentiford, Melba G. Bondad-Reantaso

**Affiliations:** 1 Scottish Association for Marine Science, Scottish Marine Institute, Oban, Argyll PA37 1QA UK; 2 Bureau of Fisheries and Aquatic Resources, Arellano Boulevard, 6000 Cebu City, Philippines; 3 Natural History Museum, Cromwell Road, London, SW7 5BD UK; 4 Centro i-mar, CeBiB and MASH, Universidad de Los Lagos, Puerto Montt, 1080000 Chile; 5 Verschuren Centre for Sustainability in Energy and Environment, Sydney, Cape Breton, NS B1M 1A2 Canada; 6 Biosecurity and One Health Research Centre, Murdoch University, Perth, WA 6150 Australia; 7 Lincoln University, 85084 Ellesmere Junction Road, Lincoln, Canterbury 7647 New Zealand; 8 Network of Aquaculture Centres in Asia-Pacific, Ladyao, Jatujak, Bangkok, 10900 Thailand; 9 Institute of Aquaculture, University of the Philippines Visayas, Miagao, Iloilo 5023 Philippines; 10 Institute of Ocean and Earth Sciences, University of Malaya, Jalan Lembah Pantai, 50603 Kuala Lumpur, Wilayah Persekutuan Malaysia; 11 State Key Laboratory of Marine Environmental Science and College of Ocean and Earth Sciences, Xiamen University, Xiamen, 361102 China; 12 Institute of Marine Fisheries and Oceanology, University of the Philippines Visayas, 5023 Miagao, Iloilo Philippines; 13 Zanzibar Seaweed Cluster Initiative, Zanzibar, Tanzania; 14 College of Marine Life Sciences, Ocean University of China, Qingdao, 266003 China; 15 ODI, London, SE1 8NJ UK; 16 Centre for Environment, Fisheries and Aquaculture Science (Cefas), Weymouth Laboratory, Weymouth, DT4 8UB Dorset UK; 17 Fisheries and Aquaculture Division, Food and Agriculture Organization of the United Nations (FAO), Rome, 00153 Italy

**Keywords:** Agriculture, Developing world

## Abstract

The rapid expansion and globalization of the seaweed production industry, combined with rising seawater temperatures and coastal eutrophication, has led to an increase in infectious diseases and pest outbreaks. Here, we propose a novel Progressive Management Pathway for improving Seaweed Biosecurity.

## The rise of seaweed cultivation

Seaweed cultivation is rapidly expanding globally. The leading region for seaweed production is Asia, although other regions (i.e., South America, Africa and Europe) have increasingly begun to cultivate selected seaweeds in response to rising global demand for a wide range of products dedicated to human consumption, such as food, cosmetics, pharmaceuticals and nutraceuticals. Other uses include agricultural fertilisers, livestock feed, biofuels, biomaterials used, for example in food packaging, and more recently the capture of atmospheric carbon^1^.

Currently, seaweed production accounts for ~51% of total global marine and coastal aquaculture production by volume, equating to nearly 35 million tonnes^2^. Exponential growth of the seaweed industry, particularly in the last 50 years, resulted in the sector reaching USD 14.7 billion in 2019^2^. Seaweeds are cultivated in over 54 countries worldwide^2^ at various scales, from less than one to many thousands of hectares^3^. The seaweed industry provides jobs to over 6 million farmers, predominantly in coastal communities in low and middle-income countries. These communities mostly sell their seaweed products to foreign, multi-national companies for processing and export^3^.

## Problems derived from a lack of seaweed biosecurity guidelines

**Fig. 1:**
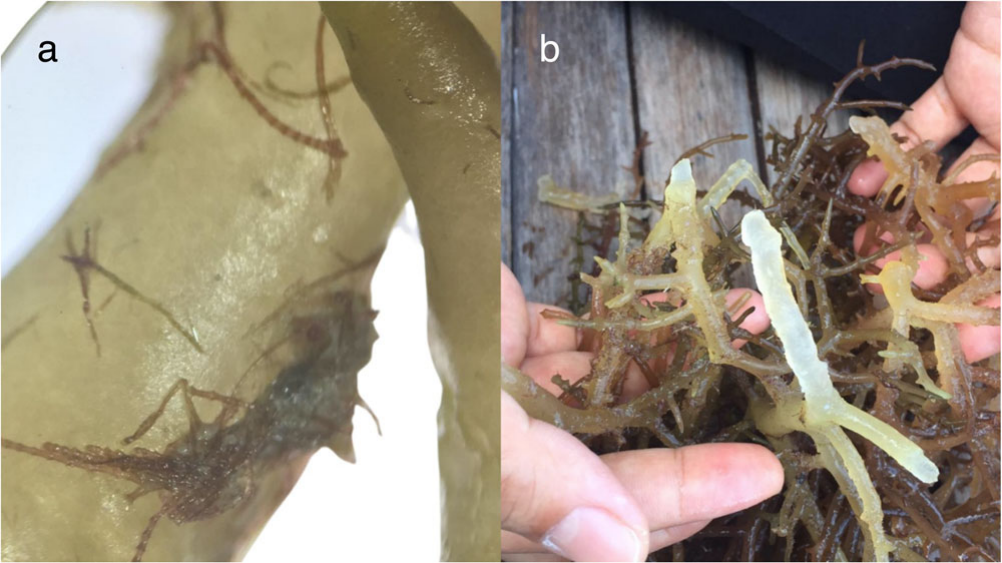
Examples of the diseases of red and brown seaweeds in aquaculture in Asia. **a** Epiphytic filamentous algae in *Kappaphycus* spp.; **b** Ice-Ice syndrome in *Kappaphycus* spp. Credit: Jennefe Cabarubias.

The rapid expansion and globalization of the seaweed industry, in combination with escalating climate change-related events, and a rise in eutrophication of coastal environments, has led to an increased prevalence of infectious disease and pest outbreaks^4^. The Philippines alone, recorded an income loss of USD 32 million between 2011–2012, due to seaweed disease outbreaks, such as Ice-Ice disease (IID) and epiphytic filamentous algae (EFA) (Fig. 1), poor quality cultivars (i.e., infected) and natural disasters^5^. In China, disease losses in *Porphyra* farming alone reached USD 410 million in 2021^4^. Similar economic losses across a broad range of seaweed species have also been seen in the Republic of Korea, Tanzania, and Indonesia^3^.

Research to identify seaweed diseases and pests, including viruses, bacteria, protists and eukaryotic endophytic algae, is ongoing^6^. However, we have only seen recent evidence of measures applied for seaweed disease prevention, treatment, and mitigation^5,7^. The control of grazers, epiphytes, and competing invertebrates, coupled with fouling algal outbreaks, which deter seaweed growth, is particularly difficult to manage. This is especially true for seaweeds grown under open sea conditions, as compared with land- or pond-based animal aquaculture production systems, where external conditions and hazards may be easier to mitigate^8,9^.

Our recent work has, however, proven the effectiveness of relatively simple biosecurity measures in Malaysia, such as the use of healthy, uninfected propagules, regular simple cleaning of the seaweed thallus and farm ropes to remove biofouling and early identification of infected stock. These measures significantly reduce the incidence of disease and epi-endophytes in red algal carrageenophytes *Kappaphycus* spp. throughout the entire cultivation period, improving both seaweed quality and market value^8^. In China, the use of bleaching powder or potassium permanganate is now routinely applied to the water supplies in seedling nurseries to prevent the growth of the main pathogenic microorganisms that are known to cause disease in the seedlings of the kelp *Saccharina japonica*^10^.

To date, primary international mechanisms for controlling exotic diseases associated with trade in aquaculture organisms and products (e.g., the Aquatic Code of the World Organisation for Animal Health (WOAH)) do not include those that impact seaweeds and aquatic plants, in that there are no international standards for the notification, diagnosis and control of diseases and pests significant to the seaweed industry. Without a clear mechanism for reporting these outbreaks from the local to the international level, they continue to occur largely under-reported in many countries. With no reporting mechanisms and evidence-based biosecurity measures in place^11^, producers typically either discard diseased and pest-infested crops into the surrounding water body or attempt to treat them using ‘unsanctioned’ methods, such as the use of inorganic fertilizers or biological growth stimulants, attempting to increase crop resistance to disease and epiphytes^5^.

In aquaculture, the lack of effective implementation and enforcement of guidelines on how to deal with infectious diseases and pests, in many cases, has led to the collapse of an industry at local and regional levels^12^. The international translocation of stock has also led to wider environmental concerns, particularly when invasive seaweeds and their associated diseases and pests have escaped and become established in the wild^6^. It is, therefore, important that the concept of biosecurity and the greater control of diseases, pests and wider environmental hazards, which can limit supply^9^, are incorporated into developing policies and practices. Such actions would protect the rapidly expanding seaweed aquaculture industry, both at national^5,7,13^ and international scales^11^.

## The Progressive Management Pathway for improving aquaculture biosecurity—seaweed (PMP/AB-Seaweed)

Whilst the dominant seaweed-producing countries (i.e., China and the Republic of Korea) have introduced biosecurity measures that have been implemented by stakeholders, most countries lack even basic measures^3^. Re-emerging or newly emerging disease and pest challenges, therefore, continue to threaten the sustainable development of this global industry^3^. We have discovered that the main reasons for the lack of measures include: (i) minimal seaweed-specific biosecurity policies at a national and international level, with the exception of China and the Republic of Korea; (ii) lack of robust evidence-based biosecurity measures; and (iii) limited guidance for implementing these measures and for the designation of responsibility to ensure the measures are followed throughout the supply chain^11^. These reasons are further exacerbated by a lack of coherent biosecurity/ hazard management across the supply chain in all sectors of the aquaculture industry. Consequently, a single actor cannot be held ‘responsible’ for losses that may occur at any given stage in the chain^9^.

The UN FAO and partners have recently developed a Progressive Management Pathway for improving Aquaculture Biosecurity (PMP/AB), to specifically address the need for strategic planning, which further guides and supports countries towards achieving sustainable aquaculture biosecurity and health management systems^14^. The PMP/AB was adapted from the “Progressive Control Pathway” (PCP) approach, which was internationally adopted for the planning and monitoring of risk mitigation strategies for the elimination, or prevention, of livestock and zoonotic diseases, such as Foot and Mouth Disease in cattle^15^. The PMP/AB is expected to play a major role in the systemic reduction of diseases and pests throughout the aquaculture industry, supported by national biosecurity tools^9,14^. We believe, however, that there is an urgent need to adapt this PMP/AB specifically for the seaweed sector. This seaweed-specific PMP/AB would provide a key role in controlling hazards associated with the industry and be part of a holistic approach to de-risking the associated supply chains^9^. The PMP/AB-Seaweed would also provide clarity for seaweed producers and government agencies across this burgeoning global industry, particularly since seaweeds are continually being debated as to whether they should be considered as a plant or animal, or whether they should come under aquatic or terrestrial jurisdiction^11^. We, therefore, propose a 4-stage Progressive Management Pathway for improving seaweed biosecurity (PMP/AB-Seaweed).

## How would the PMP/AB-Seaweed work?

**Fig. 2:**
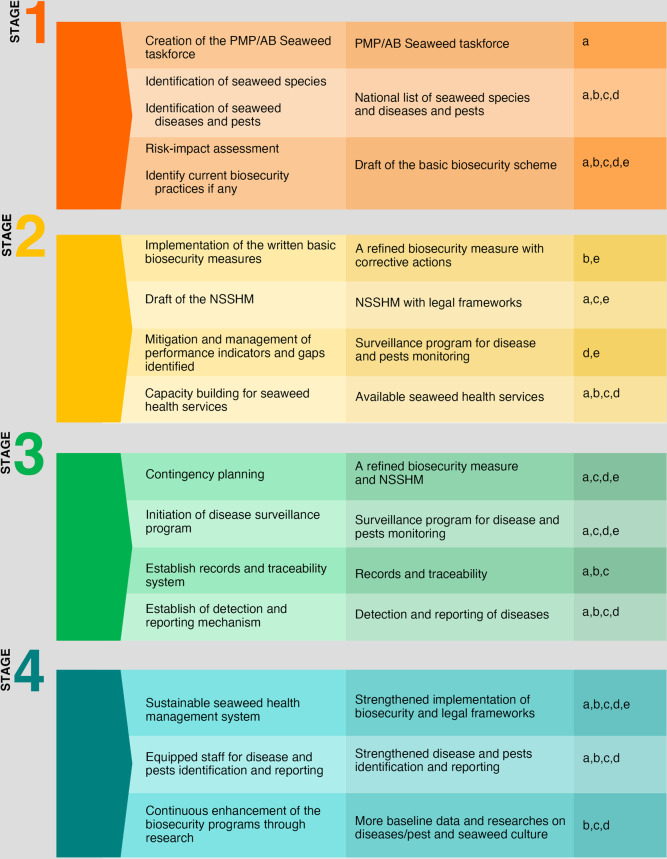
The four stages of the Progressive Management Pathway for improving Seaweed biosecurity (PMP/AB-Seaweed), including; the processes and steps, tangible outputs and constraints. (a) Technical know-how, (b) Investment capital, (c) Capacity building, (d) Innovation, (e) Inclusivity.

Each seaweed-producing country will need to produce their own PMP/AB-Seaweed, which would complement any existing or developing national strategies and biosecurity plans with relevance to the wider aquaculture sector and aquatic plant health. This pathway will be based on four key stages: (i) defining the biosecurity strategy (or risk), (ii) implementing the biosecurity systems, (iii) enhancing the biosecurity measures and preparedness, and (iv) establishing sustainable biosecurity and health management systems to support national seaweed aquaculture sectors (Fig. 2). On reaching the final stage, the country will be able to demonstrate that it has established a holistic approach to cultivating seaweeds, which not only prevents pest and disease outbreaks, but reconnects the health of humans, animals, and ecosystems in an economic and socio-political context—the One Health approach^16^.

The first stage defines the biosecurity strategy for seaweed aquaculture, through the appointment of a public-private taskforce, as recommended in the PMP/AB^14^. This taskforce will be responsible for developing a National Strategy for Seaweed Health Management (NSSHM), which would outline the strategic vision and guide investment to strengthen the seaweed health system. This type of strategy has been developed by many countries worldwide for animal health^17^, most recently in Egypt^18^. No equivalent national health strategies for seaweeds exist, however, with the exception of China^4^. For example, in Chile, even where nuisance seaweeds have been declared as pest species, no management strategies have been introduced for their control^19^. This stage will also involve the identification of the cultivated seaweed species, their diseases, pests, high risk practices throughout the supply chain and the most practical, cost-effective biosecurity measures, which target these risks. It will also promote capacity building, including regular training programs, which have proven to be invaluable, particularly for small-scale farmers with limited financial resources, for example, in the fight to control African Swine Fever in East Africa^20^.

Individuals who are actively managing seaweed farms (e.g., representative of seaweed producers), seaweed processors, Non-Government Organizations (NGOs), pathogen specialists, research scientists and the country’s Responsible Authority (e.g., focal unit in a mandated government office, such as fisheries or aquaculture), which has responsibility and competence for mandating the implementation of aquatic health measures and standards, will all be involved in this taskforce. The latter organization will typically initiate the formation of the group, since they have an in-depth knowledge of the existing aquaculture regulations and will be able to lead a series of consultations to enable the co-creation of effective seaweed-specific biosecurity policies^5,7,11,13^. The taskforce will formulate the basic biosecurity scheme, that will be aligned with international standards, meet the regulatory requirements and be practical and easy to implement by any seaweed producer. This will be irrespective of the type of operation, the size of the farm or the seaweed species produced. The biosecurity scheme will be based on existing knowledge, attitudes and practices of the farm owners and stakeholders (e.g., buyers, processors and distributors), captured using standardized interview techniques, which we have successfully trialed with over 350 seaweed farmers in the Philippines^21^, Malaysia^22^ and Tanzania^23^.

The information gained from these interviews, for example the common practice of unintentionally transferring infected seedlings between neighboring farms and the unequal access of women and men to training, will enable the taskforce to identify the critical risk points along the supply chain^8^ and to carry out an informed risk-based analysis^9^, that is inclusive of all stakeholders^24^. It will also enable the identification of the most practical, cost-effective biosecurity measures (e.g., use of healthy and disease-resistant seedlings, quarantine and disease and pest surveillance), which are commonly used by other terrestrial agricultural sectors (e.g., maize, bananas, coffee)^25^ and the seaweed industry, in the more advanced seaweed-producing countries, such as China and the Republic of Korea^3^.

The development of a key biosecurity tool—a national seaweed pathogen list, will also be led by the taskforce. This list will include diseases and pests that are, or have the potential to be yield-limiting for the industry^7^. Similar lists have been developed over recent decades, to help focus surveillance activities for terrestrial (animal and plant) and aquatic animal pathogens^26,27^. A list for seaweed pests and diseases though is well overdue, even though a number of yield-limiting diseases have been detected for over a decade^28^. A key challenge for any list of this kind, however, is to keep it updated, particularly in countries with limited resources. Novel, community-based, multi-lingual, web portals for reporting seaweed diseases and pests, for example ‘My seaweed looks weird’^29^, could provide these countries with invaluable assistance in diagnosing emergent diseases and pests and in updating their lists.

The second stage involves the implementation and modification, if necessary, of the biosecurity measures and improvements to the NSSHM. Our research has highlighted that the location and size of the farm, plus the species grown and environmental factors associated with seasonal changes can influence the efficacy of the biosecurity measures^8,30^. Thus, some modifications to the initially proposed measures and the NSSHM may need to be applied at this stage.

Ensuring compliance, through audits, certification and training is also introduced in Stage 2. Audits and certification have been routinely practiced in other aquaculture sectors for many years. Eight prevalent certification schemes currently exist for salmon aquaculture in Norway, Chile and Scotland alone^31^. They are a relatively new concept in seaweed farming, however, with a company in Korea obtaining the world’s first Aquaculture Stewardship Council- Marine Stewardship Council (ASC-MSC) certificate for the sustainable farming of seaweed in 2019^32^. The effectiveness of these audits and certification schemes has been questioned, although for many aquaculture sectors they are becoming increasingly mandatory, with companies requiring certification to trade their produce internationally^31^. For many small-scale seaweed farmers, a lack of sufficient finance, will exclude them from complying with any international standards^3^. National audit and certification schemes, however, could primarily focus on improving the farmers’ knowledge of biosecurity and their adoption of effective, low cost, farm-based biosecurity measures through participatory training events. These events could be funded by government initiatives and perhaps processors, who stand to benefit from the enhanced quality of the raw materials. The adoption of measures has remained a major challenge in other sectors, with pig farmers in Uganda requiring financial incentives to compensate for the higher costs of implementing biosecurity measures^20^. A key focus for the taskforce at this stage, therefore, will be to secure financial aid to assist small-scale farmers in engaging with their national audit and certification schemes.

The third stage will continue the implementation of the biosecurity measures, with a specific focus on strengthening contingency planning and the operation of an effective national surveillance and rapid detection system^7^. Measures for the effective control and/or suppression of exotic, endemic or emerging diseases and pests of high national concern to the seaweed sector will also be trialed and tested for their effectiveness^5,7,11,13^.

Building capacity for the rapid detection and diagnosis of seaweed diseases and pests will, therefore, be a key priority. It is likely that a national designated testing laboratory will be needed, one which may certify and report directly to the Responsible Authority for seaweed biosecurity. All nations currently have these laboratories, with varying degrees of competence for animals, plants, and aquatic organisms^33^. Very few countries, however, have specific centers for seaweed testing, with the majority reliant on specialists within academic and governmental organizations^3^. The creation of new laboratories or, for countries where there are financial constraints, the extension of existing facilities will, therefore, need to be considered at this stage. A key challenge, however, will be encouraging their use by the farmers, particularly those lacking the financial resources and/ or the ability to physically transport the diseased seaweeds to the testing laboratory, since many farms are located in remote locations. Unfortunately, these are problems which are experienced throughout the aquaculture sector globally^33^. Advances in early disease and pest diagnostic technology, however, driven by infections for terrestrial hosts, highlighted by the COVID-19 pandemic, will hopefully lead to a rapid increase in efficient, accurate and low-cost techniques, that can be applied to aquatic organisms^33^. This process will require significant commitments from key stakeholders, in terms of financial, infrastructure and human resources. By demonstrating the efficacy of simple biosecurity measures in significantly lowering the incidence of pests and disease and the subsequent improvements in yield and crop quality, as has been achieved in Malaysia and Tanzania^8,30^, stakeholders will be able to see the benefits of investing in these measures.

The fourth and final stage will involve the country having a sustainable NSSHM, fully supported by the appropriate legislation, that can effectively reduce the impacts of seaweed diseases and pests in the supply chain. Further research will be essential at this stage on the development or improvement of existing control strategies, the treatment of diseases and pests of concern and the updating of legal frameworks and audit requirements^7,11^, which will need to be supported by the taskforce and any other relevant stakeholders. Trading partners will now have the confidence to buy seedlings, fully grown seaweeds and their associated products, knowing that they will not be importing any yield-limiting diseases or pests.

## Implementing the PMP/AB-Seaweed

We are aware that implementation of the PMP/AB-Seaweed will be a challenge, particularly in countries where there is limited infrastructure, capital investment and access to markets. Given the limited wealth of many small-scale seaweed farmers, the development and implementation of the PMP/AB-Seaweed must be supported by government-led initiatives and new business models, which encourage investment, innovation and capacity building to improve biosecurity practices globally (Fig. 2). A ‘*one-size-fits all*’ approach, unfortunately will not work for this industry, so management strategies and biosecurity practices will need to be tailored to the local conditions, in particular the environmental, financial, technological and human resource capacity for each farm.

The rapid expansion and intensification of the seaweed industry are unlikely to abate soon, as the world increasingly looks toward the oceans to provide new sources of nutrition and nature-based solutions to climate change^3,34^. The timing seems right though now to introduce this PMP-AB-Seaweed, which will form an integral component of a whole supply-chain approach for de-risking aquaculture supply chains^9^. We believe it will also profoundly change the structure of this industry, ultimately allowing countries access to markets worldwide and promoting the implementation of the ‘One Health’ approach to ensure environmental, social and crop health^16^.
